# Research on the Mechanism of Low-Temperature Oxidation of Asphaltene

**DOI:** 10.3390/molecules28145362

**Published:** 2023-07-12

**Authors:** Zhengchong Zhao, Haiyang Yang, Jingjing He, Fuqiang Hu, Fan Cheng, Hai Liu, Chunli Gong, Sheng Wen

**Affiliations:** 1 School of Chemistry and Materials Science, Hubei Engineering University, Xiaogan 432000, China; 2 HuBei FTSCI BioTech Co., Ltd., Xiaogan 432000, China; 3 Hubei Engineering & Technology Research Center for Functional Materials from Biomass, Hubei Engineering University, Xiaogan 432000, China; 4 Hubei Collaborative Innovation Center for Biomass Conversion and Utilization, Hubei Engineering University, Xiaogan 432000, China

**Keywords:** asphaltene, low-temperature oxidation, hydrogen peroxide, propionic anhydride, methanol, mechanism, molecular simulation

## Abstract

Asphaltene extracted from heavy oil was oxidized by a mixture of propionic anhydride and hydrogen peroxide at a low temperature of 50 °C. Elemental analysis, infrared analysis, proton nuclear magnetic resonance analysis, and gas chromatograph/mass spectrometer analysis results indicated that oxygen addition, side chain cleavage, and condensation reactions mainly occurred in the oxidation process. The oxidation products were divided into 28% methanol solubles and 72% methanol insolubles. There were mainly fatty acids and fatty acid esters in the methanol solubles. There were also small amounts of aromatic compounds with low condensation in the methanol solubles, and the alkyl side chains were mostly short ones. The degree of aromatic ring condensation in the methanol insolubles was slightly higher than that of the pristine asphaltene. There were still some long unbroken chains in the methanol insolubles after the low-temperature reaction. The molecular dynamics simulation results show that the distribution of propionic anhydride around the asphaltene molecules can promote the oxidation of asphaltene. This low-temperature oxidation technology can be used to process asphaltenes to improve the profitability of heavy-oil-processing enterprises.

## 1. Introduction

The rapid development of the world economy has promoted sustained growth in the demand for oil. Conventional oil resources can no longer meet this demand [1]. In this case, heavy oil with high asphaltene content is gradually being developed as a kind of unconventional petroleum resource. It exhibits a large specific gravity and high viscosity. The viscosity of heavy oil increases exponentially with the increase in asphaltene content [2]. Asphaltene, as the most complex component in heavy oil, has the characteristics of a low hydrogen-carbon ratio and high average molecular weight. Asphaltene is the most difficult component to modify in heavy oil processing [3]. In the process of pyrolysis or hydrogenation of heavy oil at high temperatures, asphaltene is easy to coke and deposit, thus blocking the pipeline or catalyst channel [4,5]. In order to make full use of heavy oil resources, it is necessary to solve the problem of asphaltene upgrading.

Asphaltenes were extracted from heavy oil to study their molecular structures and reaction characteristics [6,7,8,9,10]. Asphaltenes are organic compounds with continental or archipelagic aromatic molecular structures [11,12,13]. The asphaltene aromatic core is made up of approximately seven aromatic rings [14,15]. The naphthenic rings in asphaltene molecules are mostly around aromatic rings, as deduced from observations of active hydrogen in asphaltene [16]. Alkyl side chains and alkyl bridges are the main functional groups bonded to the asphaltene aromatic cores. The molar concentration of alkyl side chains and alkyl bridges reduces as their carbon number increases. The average length of the alkyl chain is four to five carbons [17]. Chemical structures of sulfur in petroleum asphaltenes are mainly sulfur ether and thiophene [18]. Both pyridinic-N and pyrrolic-N are present in asphaltenes [19]. The oxygen-containing groups are mainly acids and phenols [20]. Asphaltene pyrolysis or hydrogenation processes need to be carried out at high temperatures, which can easily lead to asphaltene coking [21]. Oxidized asphaltene can be carried out at a lower temperature [22], and this technology is a promising, low-cost, effective, and environmentally friendly technology for asphaltene upgrading. The asphaltenes were adsorbed on the nanocatalyst by thermogravimetric analysis to investigate the oxidation effect above 200 °C [23,24,25,26,27]. The asphaltenes were oxidized by air with a metal oxide nanocatalyst to produce light components or CO_2_. However, this kind of catalytic reaction often introduces metal elements, which hinders subsequent processing. Sodium hypochlorite not only can oxidize sp^3^ hybridized carbon in aromatic compounds but also can oxidize some sp^2^ hybridized carbon at 40–70 °C [28,29]. However, it has low oxidation selectivity for aromatic carbon [30] and may cause environmental pollution due to chlorine content. The ruthenium ion catalytic oxidation (RICO) method can open aromatic rings in asphaltene molecules at room temperature [31,32,33]. The main catalyst component in this method is RuO_4_, which has high selectivity for sp^2^ hybridized aromatic carbons. RICO is only used for the analysis of asphaltene molecular structures due to the high cost of catalysts. By generating ·OH radicals, H_2_O_2_ can break the bridge bond between methylene and ether in the structure of organic macromolecules to degrade organic macromolecules [34]. Using hydrogen peroxide and acetic anhydride as oxidants, Wang [35] decomposed coal at 50 °C to produce dicarboxylic acid. Oxidizing asphaltene with hydrogen peroxide and propionic anhydride at a low temperature is a low-cost, safe, and environmentally friendly upgrading technology.

To efficiently utilize asphaltene resources without introducing metal-containing catalysts, the low-temperature oxidation mechanism of asphaltenes was studied at 50 °C using hydrogen peroxide and propionic anhydride as oxidants.

## 2. Results and Discussion

Approximately 0.5 g of asphaltene powder was reacted with propionic anhydride and hydrogen peroxide at 50 °C for 24 h, and the oxidized products were separated into MeOHS and MeOHI. Table 1 shows the dosage of the reagents and the amount of product generated in the asphaltene oxidation experiment.

Table 1 shows that the total mass of asphaltene after oxidation is 0.6956 g. There is an increase of 26% compared with 0.5520 g of pristine asphaltene. MeOHS and MeOHI accounted for 28% and 72% of the oxidation products, respectively.

### 2.1. Elemental Analysis

The elemental compositions and atomic masses of pristine asphaltene, MeOHS, and MeOHI after the reaction are shown in Table 2.

It can be seen from Table 1 and Table 2 that the content of S decreased from 0.0349 g before the oxidation to 0.0291 g after the oxidation of asphaltene, indicating that the oxidation reaction removed some of the S. Chemical structures of sulfur in petroleum asphaltenes are mainly sulfur thioether and thiophene. The thioether and thiophene were oxidized to form sulfoxides and sulfones [36,37]. A small amount of sulfone produces SO_2_. With the volatilization of SO_2_, the sulfur content in the oxidation products decreased.

The nitrogen content decreased from 0.0073 g before the oxidation to 0.0065 g after the oxidation of asphaltene. Due to the low nitrogen content, the mass value may fluctuate within the margin of error. Therefore, the nitrogen content is considered to be almost unchanged before and after oxidation. During the oxidation process, nitrogen-containing groups are oxidized to form nitrogen oxides, but small molecules of nitrogen-containing gases cannot be formed [38]. Therefore, the nitrogen content did not change before and after the oxidation reaction.

The hydrogen content changed from 0.0337 g to 0.0345 g. The hydrogen content is almost unchanged before and after oxidation. During the reaction process, water may be generated, resulting in a decrease in H content, but at the same time propionic anhydride may also participate in the reaction, resulting in an increase in H content. Considering the two factors, the H content did not change much before and after the oxidation reaction.

The H/C atomic ratios of pristine asphaltene, MeOHS, and MeOHI were 0.87, 1.50, and 0.79, respectively. The higher the H/C atom ratio, the larger the carbon proportion of sp^3^ hybrid, and the lower the carbon proportion of sp^2^ hybrid. In the molecular structure of asphaltene, sp^3^ hybrid carbon is distributed mainly in the alkyl side chain and naphthenic ring, and sp^2^ hybrid carbon is mainly aromatic carbon. When aromatic hydrocarbons are oxidized, alkyl side chains are more likely to oxidize and produce fatty acids [39,40]. The H/C atomic ratio of MeOHS was larger than that of pristine asphaltene, indicating that MeOHS contained fatty acids generated by the cleavage of alkyl side chains and oxidation products with a small quantity of aromatic rings. The H/C atomic ratio of MeOHI was smaller than that of pristine asphaltene, indicating that MeOHI contained more condensation reaction products.

The O/H and O/C atomic ratios of MeOHS and MeOHI were both larger than that of pristine asphaltene, indicating that the oxidation reactions occurred more extensively [41]. The O/H atomic ratio of MeOHS was more than two times that of MeOHI, and the O/C atomic ratio of MeOHS was approximately four times that of MeOHI. It indicated that the proportion of oxygen-containing groups was high and that they were easier to dissolve in methanol.

Asphaltene is the key component that causes the high viscosity of heavy oil. The viscous nature of asphaltenes is an important obstacle to the recovery and processing of heavy oil. The oxygen content of asphaltene increases greatly after oxidation. This will result in the enhancement of asphaltene-induced viscosity. If alkali is added in the process of heavy oil extraction or oxidation processing, the viscosity of oxidized heavy oil will be reduced below the initial value [42]. In this way, viscosity reduction is achieved [43]. Asphaltene oxidation can be applied not only to heavy oil processing, but also to heavy oil recovery.

### 2.2. Nuclear Magnetic Spectrum Analysis

**Figure 1:**
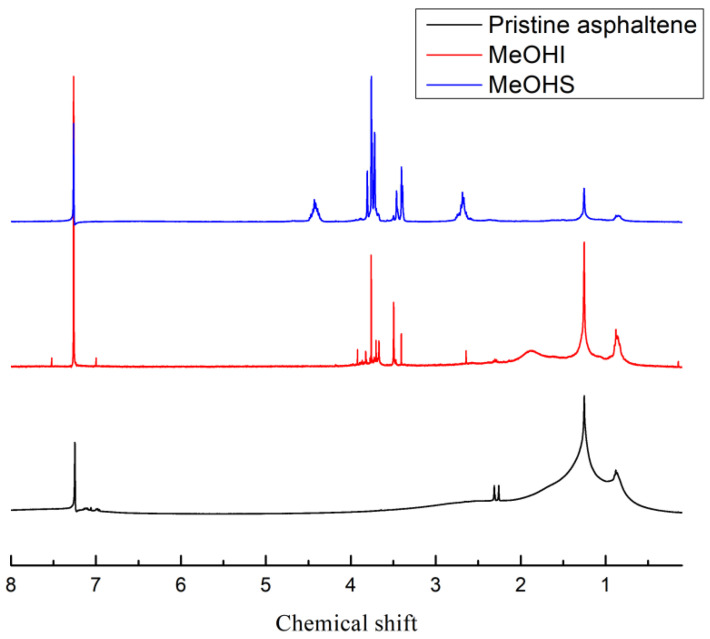
^1^H NMR Spectra of pristine asphaltene, MeOHS, and MeOHI.

The ^1^H NMR spectra of pristine asphaltene, MeOHS, and MeOHI with deuterated chloroform as solvent are shown in Figure 1. The nuclear magnetic resonance spectroscopy data are shown in Table 3.

In the NMR spectrum, the integral area is proportional to the corresponding number of atoms. The proton resonance between 10.0 ppm and 6.0 ppm is thought to be caused by the aromatic protons (H_A_). Hydrogens in α-position to aromatic ring (H_α_), CH_2_ and CH hydrogens other than in α-position to aromatic ring (H_β_), and terminal (t-) CH_3_ hydrogens other than in α-position on aliphatic chain (H_γ_) have resonances between 4.0 ppm and 2.1 ppm, 2.1 ppm and 1.0 ppm, and 1.0 ppm and 0.4 ppm, respectively. The C/H atomic ratio data come from elemental analysis in Section 2.1. Total hydrogens (H_T_) are calculated from Formula (1). Total carbons (C_T_) are equal to H_T_ times C/H atomic ratio. Aromatic rates (f_A_) refers to the ratio of aromatic carbon to the total amount of carbon in a molecular structural unit. Aromatic rates (f_A_) are calculated from Formula (2). Peripheral hydrogen substitution rates of aromatic rings (σ) are calculated from Formula (3). Aromatic ring condensation degree parameters (H_AU_/C_A_) are the ratios of the hydrogen assumed not to be replaced in the aromatic portion or by which the aromatic nucleus might be replaced to aromatic carbon atoms. They are calculated from Formula (4). In the case of the same aromatic ring, the smaller the H_AU_/C_A_, the tighter the molecular structure of asphaltene. Branching indexes of alkyl side chains (BI) are calculated from Formula (5) [44].

(1) $$H_{T}=H_{A}+H_{\alpha }+H_{\beta }+H_{\gamma }$$

(2) $$f_{A}=\frac{C_{T}/H_{T}-(H_{\alpha }+H_{\beta }+H_{\gamma })/2H_{T}}{C_{T}/H_{T}}$$

(3) $$\sigma =\frac{H_{\alpha }/2}{H_{A}+H_{\alpha }/2}$$

(4) $$\frac{H_{\mathrm{AU}}}{C_{A}}=\frac{H_{A}/H_{T}+H_{\alpha }/2H_{T}}{C_{T}/H_{T}-(H_{\alpha }+H_{\beta }+H_{\gamma })/2H_{T}}$$

(5) $$\mathrm{BI}=\frac{H_{\gamma }}{H_{\beta }}$$

It can be seen from Table 3 that the values of aromatic rates f_A_ of the pristine asphaltene, MeOHS, and MeOHI were 0.64, 0.42, and 0.70, respectively. The value of aromatic rate f_A_ of the MeOHS was smaller than that of the pristine asphaltene, indicating that the proportion of aromatic carbon in the MeOHS was small [45,46,47]. In contrast, the aromatic rate f_A_ of MeOHI was larger than that of pristine asphaltene, indicating that aromatic carbon accounted for a large proportion of MeOHI. Combined with the previous mass analysis, the total aromatic carbon mass increased by approximately 0.08 g after the oxidation of asphaltene. In other words, the total aromatic carbon mass increased by approximately 9% after the oxidation of asphaltene. The aromatic carbon content did not decrease, but increased. This indicates that the oxidant has a very low selectivity for the oxidation of aromatic carbon. The peripheral hydrogen substitution rate of the aromatic rings (σ) of MeOHS was higher than that of pristine asphaltene, indicating that some aromatic hydrogen of MeOHS may have been substituted by hydroxyl or carboxyl groups. The H_AU_/C_A_ value of MeOHS was much larger than that of pristine asphaltene, indicating that the degree of aromatic ring condensation in MeOHS was very low. The percentage of H_α_ of the pristine asphaltene, MeOHS, and MeOHI accounted for 18.5%, 53.5%, and 19.1% of the total hydrogen, respectively. The percentage of hydrogen H_β_ of the pristine asphaltene, MeOHS, and MeOHI accounted for 45.7%, 17.4%, and 43.8% of the total hydrogen, respectively. The percentage of H_α_ in MeOHS was high, whereas the percentage of H_β_ was small, indicating that the alkyl side chains in MeOHS were mostly short side chains. The percentage of hydrogen H_β_ in MeOHI was high, which is likely because there were still some long unbroken chains at low reaction temperatures.

### 2.3. Infrared Spectroscopic Analysis

**Figure 2:**
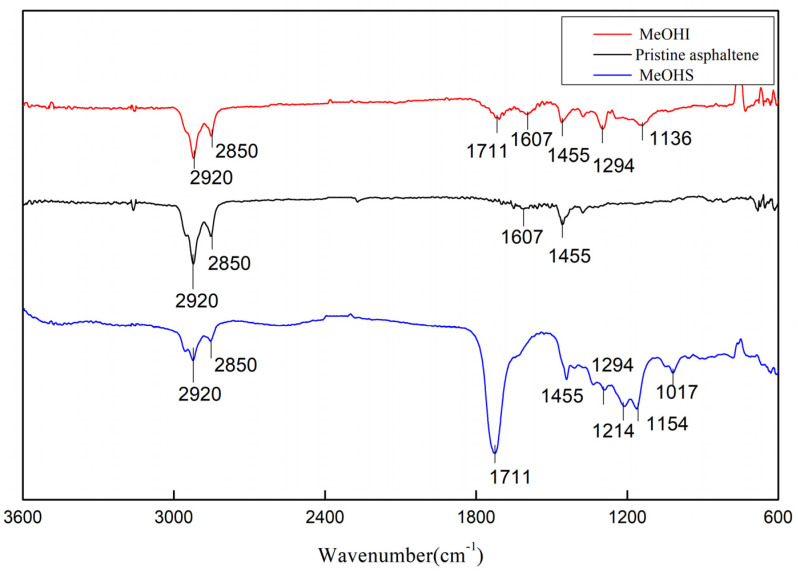
The infrared spectra of pristine asphaltene, MeOHS, and MeOHI after the oxidation reaction: the black line indicates asphaltene, the red line represents MeOHI, and the blue line shows MeOHS.

Infrared spectra of pristine asphaltene, MeOHS, and MeOHI after the oxidation reaction are shown in Figure 2.

It can be seen from the infrared spectra that the peaks near 2920, 2850, and 1455 cm^−1^ are assigned to methylene and methyl groups. There is an obvious strong peak near 1711 cm^−1^ in the spectra of both MeOHS and MeOHI. The peak belongs to the characteristic absorption peak of the carboxyl group (C=O). The peak near 1294 cm^−1^ in the spectra of both MeOHS and MeOHI, which belongs to primary hydroxyl or secondary alcohol, was also significantly enhanced.

The peaks near 1214 cm^−1^ and 1154 cm^−1^ of MeOHS are assigned to phenolic hydroxyl groups. The peak near 1017 cm^−1^ of MeOHS is attributed to the C-O bending vibration of primary alcohol.

The peak near 1607 cm^−1^ of MeOHI is attributed to the C=C stretching vibration of the aromatic ring [48]. The peak near 1136 cm^−1^ of MeOHI is assigned to tertiary alcohol.

Comparing the IR spectra of the pristine asphaltene, MeOHS, and MeOHI, those enhanced infrared peaks indicate that oxidation reactions have taken place. And the oxygen-containing groups formed by oxidation include primary alcohol groups, phenolic hydroxyl groups, carboxyl groups, and tertiary alcohol groups.

### 2.4. GC/MS Analysis of MeOHS

**Figure 3:**
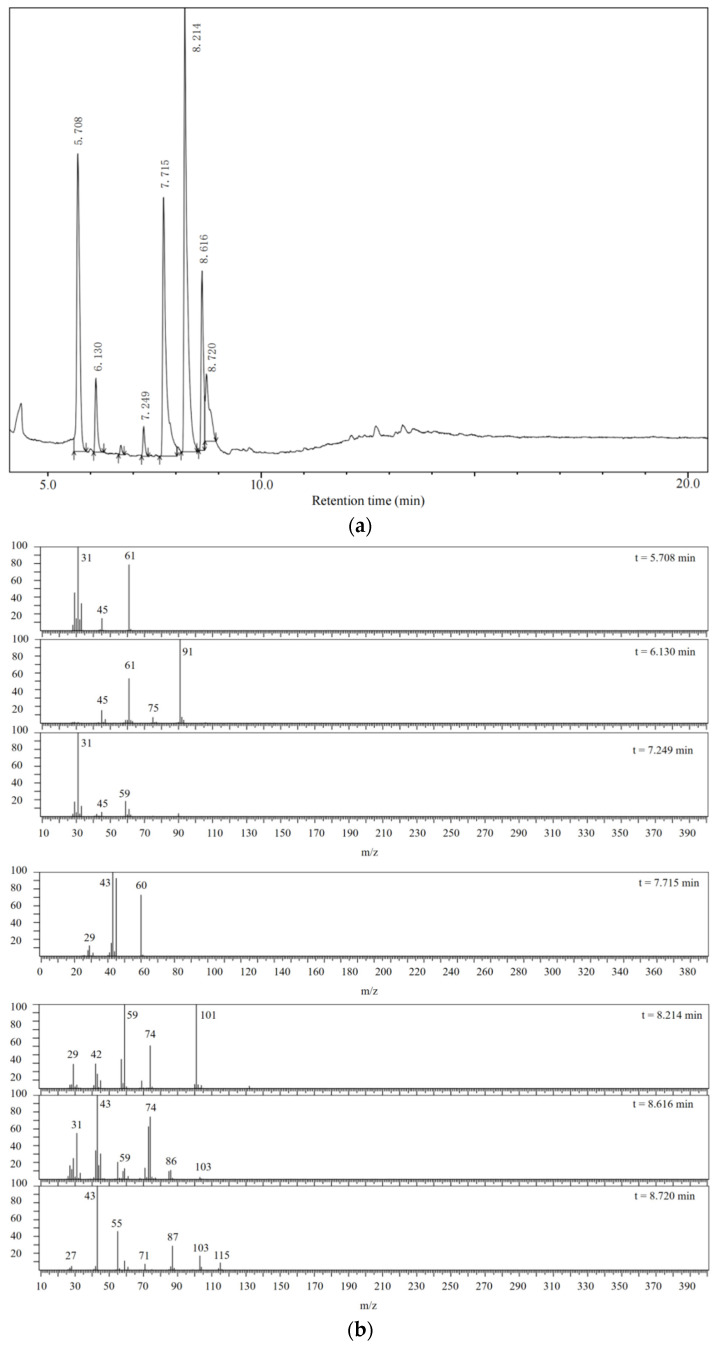
Gas chromatogram and mass spectra of MeOHS. (**a**) Gas chromatogram of MeOHS; (**b**) Mass spectra of components with different retention times.

The chemical composition of MeOHS was analyzed with a Shimadzu gas chromatograph/mass spectrometer (GC/MS). The GC/MS spectra are shown in Figure 3.

In total, seven kinds of compounds were detected with GC/MS. They are 1,3,5-trioxane, 2-(methylsulfonylmethylsulfanyl) ethanol, methyl 2-hydroxyacetate, acetic acid, dimethyl propanedioate, methyl 3-hydroxypropanoate, and pentan-2-yl acetate, as shown in Table 4. The detected substances are mainly esters, which contain carbonyl groups and primary alcohol hydroxyl groups. This corresponds to the infrared analysis results of MeOHS. Among the seven products, acetic acid and dimethyl propanedioate accounted for more than 56%. This indicates that propionic anhydride was probably involved in the oxidation to form acetic acid and dimethyl propanedioate. There are also some compounds with larger molecular weights whose structures have not been detected, according to Figure 3a.

### 2.5. Molecular Dynamics Simulation Analysis

**Figure 4:**
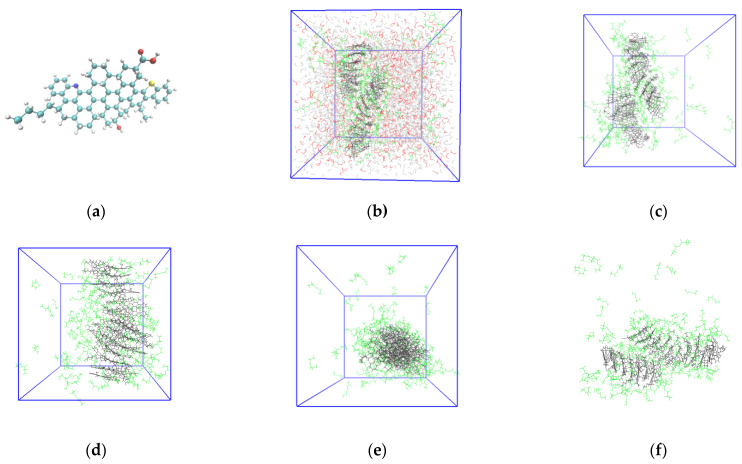

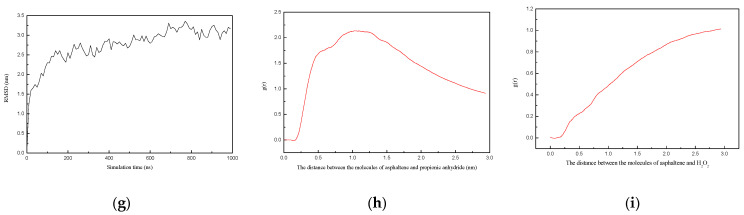
Equilibrium system of asphaltene, propionic anhydride, hydrogen peroxide, and water. (**a**) Molecular model of asphaltenes containing white hydrogen, blue nitrogen, red oxygen, cyan carbon, and yellow sulfur; (**b**) the equilibrium system containing black asphaltene, green propionic anhydride, red hydrogen peroxide, and silver water; (**c**–**f**) are different perspectives of the equilibrium system that hides hydrogen peroxide and water; (**g**) root mean square deviation (RMSD) of the system; (**h**) radial distribution curve between the molecules of asphaltene and propionic anhydride; (**i**) radial distribution curve between the molecules of asphaltene and hydrogen peroxide.

A periodic system consisting of 7 asphaltene molecules [49], 138 propionic anhydride molecules, 881 hydrogen peroxide molecules, and 3885 water molecules was built and optimized by molecular dynamics simulation. The asphaltene molecular model and the stable equilibrium system are shown in Figure 4.

As shown in Figure 4, with increasing simulation time, the RMSD value stabilizes at approximately 3 nm, which indicates that the system has reached the equilibrium state. The asphaltene molecules are all clustered together, and the propionic anhydride molecules are distributed around the asphaltene aggregates. According to the radial distribution curve between the molecules of asphaltene and propionic anhydride, propionic anhydride molecules are mainly distributed at approximately 1 nm of asphaltene molecules. According to the radial distribution curve between the molecules of asphaltene and hydrogen peroxide, the hydrogen peroxide molecules do not disperse around the asphaltene molecules. This indicates that the molecular interaction between asphaltene and hydrogen peroxide is not as strong as that between asphaltene and propionic anhydride. Without the addition of propionic anhydride, there is not sufficient contact between asphaltenes and oxidants. This indirectly reflects that propionic anhydride can promote the oxidation of asphaltenes.

### 2.6. Oxidative Reaction Mechanism Analysis

**Figure 5:**
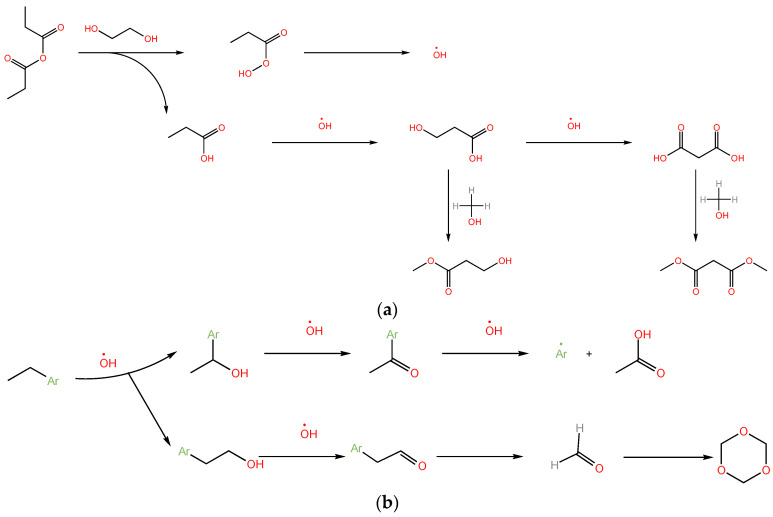
Oxidative reaction mechanism of asphaltene. (**a**) The possible formation routes of hydroxyl radicals, methyl 3-hydroxypropanoate, and dimethyl propanedioate; (**b**) The possible formation routes of acetic acid and 1,3,5-trioxane. Ar stands for aromatic rings.

The possible reaction routes of asphaltene with propionic anhydride and hydrogen peroxide are shown in Figure 5. When propionic anhydride is mixed with hydrogen peroxide, peroxypropionic acid is formed, and then hydroxyl radicals are formed. Hydroxyl radicals are extremely oxidizing species that play a leading role in the oxidation reaction. When oxidizing aromatic compounds, hydroxyl radicals mainly attack the side chains of aromatic rings [50] but also attack aromatic rings containing hydrogen atoms [35]. Asphaltenes are oxidized to form aldehydes, fatty acids, and alcohols [51]. Fatty acids and alcohols may react to form esters. Formaldehyde may undergo a polymerization reaction to form 1,3,5-trioxane [52]. These fatty acids, fatty acid esters, and trimaldehyde can be used as chemical raw materials [53]. These chemical raw materials are more valuable than asphaltenes. MeOHI after oxidation of asphaltene has its unique properties due to its high oxygen content, more aromatic condensation components, and larger molecular weight. It can be used as an adsorbent or something else. Therefore, low-temperature oxidation of asphaltene can be used in heavy oil processing.

## 3. Materials and Methods

### 3.1. Experimental Materials and Reagents

In this study, heptane insoluble and xylene soluble fraction was extracted from the Xinjiang heavy oil of China as pristine asphaltene. The heptane insoluble substance was extracted from heavy oil by n-heptane to remove the saturated, aromatic, and colloidal components. The heptane insoluble was extracted with xylene to remove the particles and a small amount of xylene insoluble, and asphaltene was obtained after drying.

Heptane was analytically pure n-heptane. Xylene was an analytically pure mixture of xylene isomer and ethylbenzene purchased from Shanghai Aladdin Bio-Chem Technology Co., Ltd., Shanghai, China. Propionic anhydride and methanol used in the oxidation process were analytical pure reagents, and the mass concentration of hydrogen peroxide is 30 wt.%. Methanol was provided by HuBei FTSCI BioTech Co., Ltd., Xiaogan, China.

### 3.2. Asphaltene Oxidation Experimental Methods

**Figure 6:**
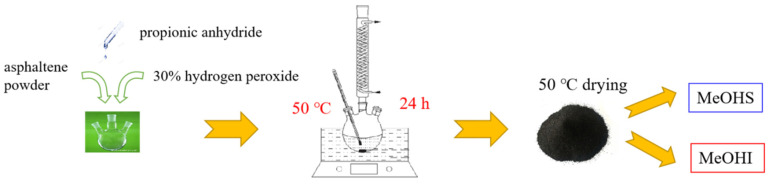
Diagram of asphaltene oxidation experiment.

The method of asphaltene oxidation is shown in Figure 6. The asphaltene initially contained large particles, which need to be finely ground with an agate mortar to facilitate adequate contact between the asphaltene and the oxidant during the oxidation reaction. Approximately 0.5 g asphaltene powder and 6 g propionic anhydride were poured into a three-necked flask. They were mixed well with magnetic stirring in a water bath. Then, about 30 mL 30 wt.% hydrogen peroxide was added to enable the asphaltene to be fully oxidized. The mixture was heated to 50 °C under liquid reflux and reacted for 24 h. The mixture was transferred to a beaker after oxidation. The residue in the three-mouth flask was washed into a beaker with methanol. Then, the mixture was dried in a blast oven at 50 °C for 24 h to remove methanol, water, excess propionic anhydride, and hydrogen peroxide. The dried residue was mixed with methanol and then dispersed by ultrasonic vibration. Then, they were separated into methanol solubles (MeOHS) and methanol insolubles (MeOHI) by centrifugal method. Finally, MeOHS and MeOHI were dried in a 50 °C oven to remove methanol. Separating the oxidation product of asphaltene into MeOHS and MeOHI can be used as a method for judging the oxidation effect [54].

### 3.3. Analysis Methods

The C, H, S, N, and O components of pristine asphaltene, MeOHS, and MeOHI were analyzed by a fully automatic VARIO EL III instrument. The content data of C, H, S, and N are directly measured by the machine. The oxygen content is calculated by subtracting the mass of the four elements CHSN from the total mass.

Proton nuclear magnetic resonance (^1^H-NMR) before and after oxidation of asphaltene was analyzed using a Bruker Ascend 700 M nuclear magnetic resonance spectrometer with deuterated chloroform (CDCl_3_) as solvent.

Infrared spectra (IR) of pristine asphaltene, MeOHS, and MeOHI were analyzed by a Nicolet 6700 Fourier transform infrared spectrophotometer.

The chemical composition of MeOHS was analyzed with a Shimadzu gas chromatograph/mass spectrometer (GC/MS).

### 3.4. Molecular Dynamics Simulation Methods

MD simulations were carried out with Gromacs software [55]. The simulation parameters were selected from the Optimized Potentials for Liquid Simulations-All Atoms (OPLS-AA) force field [56,57]. The asphaltene molecular model was derived from the molecular structure analysis of heavy oil asphaltene from Xinjiang, China [58]. The periodic models were built using Packmol software [59]. Visual Molecular Dynamics (VMD) software was selected as visualization software for structural analysis [60].

The MD simulations involved the following steps [61]: (i) the periodic model was built with 7 asphaltene molecules, 3885 spc/e water molecules, 881 hydrogen peroxide molecules, and 138 propionic anhydride molecules; (ii) it was optimized by energy minimization; (iii) MD simulations at constant pressure and constant temperature (NPT) were carried out at 323.15 K and 0.1 MPa for 20 ns; (iv) MD simulations in the canonical ensemble (NVT) were carried out at 323.15 K for 10 ns to obtain an equilibrium system.

## 4. Conclusions

Oxygen addition, side chain cleavage, and condensation reactions mainly occurred in the oxidation process of asphaltene with hydrogen peroxide and propionic anhydride at the low temperature of 50 °C. Oxidation products were divided into 28% MeOHS and 72% MeOHI. The H/C and O/C atomic ratios of MeOHS were higher than those of pristine asphaltene. There were mainly fatty acids and fatty acid esters in the MeOHS. The degree of aromatic ring condensation in MeOHS was very low, and the alkyl side chains were mostly short side chains. The H/C atomic ratio of MeOHI was smaller than that of pristine asphaltene, whereas the O/C atomic ratio was higher. The oxygen content of MeOHI was lower than that of MeOHS. The degree of aromatic ring condensation in MeOHI was slightly higher than that of pristine asphaltene. There were still some long unbroken chains in MeOHI at low reaction temperatures.

This low-temperature asphaltene oxidation technology can be used in the processing of heavy oil with high asphaltene content to increase the utilization efficiency of oil resources. The technology can also be used to in situ upgrade heavy oil in the underground without additional heating and without the introduction of metal elements that hinder downstream heavy oil processing. In short, this technology can facilitate the recovery and processing of heavy oil.

## Figures and Tables

**Table 1:** Reagent dosage and production in hydrogen peroxide–propionic anhydride oxidation test of asphaltene.

	Pristine Asphaltene	30 wt.% Hydrogen Peroxide	Propionic Anhydride	MeOHS	MeOHI
Dosage	0.5520 g	30 mL	6.9 g	—	—
Production	—	—	—	0.1974 g	0.4982 g

**Table 2:** Elemental composition and atomic mass before and after the oxidation reaction.

	Elemental Composition, wt.%					Atomic Ratio		
	C	H	S	N	O	H/C	O/C	O/H
before oxidation								
asphaltene, wt.%	84.70	6.11	6.33	1.32	1.54	0.87	0.014	0.016
after oxidation								
MeOHS, wt.%	45.16	5.60	1.83	0.65	46.76	1.50	0.78	0.52
MeOHI, wt.%	71.13	4.70	5.12	1.05	18.00	0.79	0.19	0.24

**Table 3:** Nuclear magnetic resonance spectroscopic analysis of pristine asphaltene, MeOHS, and MeOHI.

	Pristine Asphaltene	MeOHS	MeOHI
Relative value of integral area of NMR spectrum			
Aromatic hydrogens (H_A_)	0.16	0.19	0.22
Hydrogens in α-position to aromatic ring (H_α_)	0.17	0.46	0.17
CH_2_ and CH hydrogens other than in α-position to aromatic ring (H_β_)	0.42	0.15	0.39
Terminal (t-) CH_3_ hydrogens other than in α-position on aliphatic chain (H_γ_)	0.17	0.06	0.11
C/H atomic ratio	1.15	0.67	1.27
Total hydrogens (H_T_)	0.92	0.86	0.89
Total carbons (C_T_)	1.06	0.58	1.13
Aromatic rate (f_A_)	0.64	0.42	0.70
Aromatic carbons (C_A_)	0.68	0.24	0.80
Peripheral hydrogen substitution rate of aromatic rings (σ)	0.35	0.55	0.28
Aromatic ring condensation degree parameters (H_AU_/C_A_)	0.36	1.74	0.38
Branching index of alkyl side chains (BI)	0.40	0.40	0.28

**Table 4:** Organic compounds detected in MeOHS by GC/MS analysis.

No.	Retention Time, min	Peak Area of Gas Chromatography, %	Corresponding Compounds	Molecular Structures
1	5.708	21.54	1,3,5-trioxane	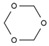
2	6.130	3.98	2-(methylsulfonylmethylsulfanyl) ethanol	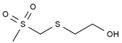
3	7.249	1.45	methyl 2-hydroxyacetate	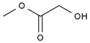
4	7.715	22.05	acetic acid	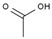
5	8.214	34.02	dimethyl propanedioate	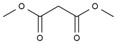
6	8.616	10.03	methyl 3-hydroxypropanoate	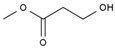
7	8.720	6.93	pentan-2-yl acetate	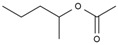
Total		100		

## Data Availability

Data available on request. The data presented in this study are available on request from the corresponding author.

## Sample Availability

Not available.
